# The effects of vitamin D and omega-3 fatty acids co-supplementation on biomarkers of inflammation, oxidative stress and pregnancy outcomes in patients with gestational diabetes

**DOI:** 10.1186/s12986-017-0236-9

**Published:** 2017-12-28

**Authors:** Maryamalsadat Razavi, Mehri Jamilian, Mansooreh Samimi, Faraneh Afshar Ebrahimi, Mohsen Taghizadeh, Reza Bekhradi, Elahe Seyed Hosseini, Hamed Haddad Kashani, Maryam Karamali, Zatollah Asemi

**Affiliations:** 1 Department of Gynecology and Obstetrics, School of Medicine, Ardabil University of Medical Sciences, Ardabil, Iran; 2 Endocrinology and Metabolism Research Center, Department of Gynecology and Obstetrics, School of Medicine, Arak University of Medical Sciences, Arak, Iran; 3 Department of Gynecology and Obstetrics, School of Medicine, Kashan University of Medical Sciences, Kashan, Iran; 4 Research Center for Biochemistry and Nutrition in Metabolic Diseases, Kashan University of Medical Sciences, PO Box 8715988141, Kashan, Iran; 5 Barij Medicinal Plants Research Center, Kashan, Iran; 6 Anatomical Sciences Research Center, Kashan University of Medical Sciences, Kashan, Iran; 7 Department of Gynecology and Obstetrics, School of Medicine, Iran University of Medical Sciences, Tehran, Iran

**Keywords:** Vitamin D, Omega-3 fatty acids, Supplementation, Gestational diabetes, Pregnancy outcomes

## Abstract

**Background:**

This study was carried out to determine the effects of vitamin D and omega-3 fatty acids co- supplementation on biomarkers of inflammation, oxidative stress and pregnancy outcomes in gestational diabetes (GDM) patients.

**Methods:**

This randomized, double-blind, placebo-controlled trial was conducted among 120 GDM women. Participants were randomly divided into four groups to receive: 1) 1000 mg omega-3 fatty acids containing 180 mg eicosapentaenoic acid (EPA) and 120 mg docosahexaenoic acid (DHA) twice a day + vitamin D placebo (*n* = 30); 2) 50,000 IU vitamin D every 2 weeks + omega-3 fatty acids placebo (n = 30); 3) 50,000 IU vitamin D every 2 weeks + 1000 mg omega-3 fatty acids twice a day (n = 30) and 4) vitamin D placebo + omega-3 fatty acids placebo (n = 30) for 6 weeks.

**Results:**

Subjects who received vitamin D plus omega-3 fatty acids supplements compared with vitamin D, omega-3 fatty acids and placebo had significantly decreased high-sensitivity C-reactive protein (−2.0 ± 3.3 vs. -0.8 ± 4.4, −1.3 ± 2.4 and +0.9 ± 2.7 mg/L, respectively, *P* = 0.008), malondialdehyde (−0.5 ± 0.5 vs. −0.2 ± 0.5, −0.3 ± 0.9 and +0.5 ± 1.4 μmol/L, respectively, *P* < 0.001), and increased total antioxidant capacity (+92.1 ± 70.1 vs. +55.1 ± 123.6, +88.4 ± 95.2 and +1.0 ± 90.8 mmol/L, respectively, *P* = 0.001) and glutathione (+95.7 ± 86.7 vs. +23.0 ± 62.3, +30.0 ± 66.5 and −7.8 ± 126.5 μmol/L, respectively, P = 0.001). In addition, vitamin D and omega-3 fatty acids co-supplementation, compared with vitamin D, omega-3 fatty acids and placebo, resulted in lower incidences of newborns’ hyperbilirubinemiain (*P* = 0.037) and newborns’ hospitalization (*P* = 0.037).

**Conclusion:**

Overall, vitamin D and omega-3 fatty acids co-supplementation for 6 weeks among GDM women had beneficial effects on some biomarkers of inflammation, oxidative stress and pregnancy outcomes.

## Background

Gestational diabetes mellitus (GDM) is impaired glucose tolerance and insulin resistance with first onset during pregnancy [1]. It affect approximately 5% of all pregnancies worldwide [2]. The its prevalence in US [3] and Iran [4] was reported 5.8 and 4.7%, respectively. GDM is associated with serious maternal and fetal complications including pre-eclampsia, fetal macrosomia, shoulder dystocia, neonatal hypoglycemia and maternal complications [5]. In addition, a 7-fold increased risk for developing type 2 diabetes mellitus (T2DM) was reported in GDM women [6]. Increased inflammatory factors contribute to pregnancy-induced insulin resistance and the development of glucose intolerance [7]. Hyperglycemia in GDM increases oxidative stress through several molecular mechanisms including the activation of protein kinase C and increased reactive oxygen species production in mitochondria [8].

Previous studies have reported that vitamin D [9] and omega-3 fatty acids levels [10] were low in GDM patients than those healthy pregnant women. Earlier, the beneficial effects of single supplementation with vitamin D or omega-3 fatty acids on biomarkers of inflammation, oxidative stress and pregnancy outcomes in GDM women were reported [11, 12]. We are aware of no study that examined the effect of vitamin D or omega-3 fatty acids co-supplementation on inflammatory factors, biomarkers of oxidative stress and pregnancy outcomes in GDM. Nowadays, there is a growing interest to use vitamin D and omega-3 fatty acids during pregnancy especially in GDM women. In a study by Mojibian et al. [13], it was observed that supplementation with 50,000 IU vitamin D every 2 weeks to women with gestational age 12–16 weeks until delivery decreased the incidence of GDM, but did not affect the incidence of pre-eclampsia, gestational hypertension, preterm labor, and low birth weight. Furthermore, in a meta-analysis study, omega-3 fatty acids supplementation during pregnancy was associated with the reduced risk of preterm delivery and improved size of the newborn [14]. However, docosahexaenoic acid (DHA) supplementation of 800 mg/day in the second half of pregnancy could not reduce the risk of GDM or pre-eclampsia [15].

There are speculations that vitamin D and omega-3 fatty acids may improve biomarkers of inflammation and oxidative stress, and pregnancy outcomes due to their effects on maternal metabolic profiles [16], increased metabolism of bile acids [17] and inhibiting the activation of nuclear factor-κB (NF-кB) [18]. This study was, therefore, carried out to determine the effects of vitamin D and omega-3 fatty acids co-supplementation on biomarkers of inflammation (primary outcomes), oxidative stress and pregnancy outcomes (secondary outcomes) in GDM women.

## Methods

### Trial design and participants

The present study, registered in the Iranian website for registration of clinical trials (http://www.irct.ir: IRCT201701305623N106), was a 6-week randomized double-blind placebo controlled clinical trial that was carried out among 120 GDM women at 24–28 weeks’ gestation, aged 18–40 years without prior diabetes diagnosed based on the American Diabetes Association guidelines [19] from September 2016 to March 2017. This study was approved by the research ethics committee of Ardabil University of Medical Sciences (AUMS) and written informed consent was taken from all patients. Exclusion criteria were taking omega-3 fatty acids supplements, insulin therapy, placenta abruption, pre-eclampsia, eclampsia, hypo and hyperthyroidism, and smokers.

### Study design

At first, participants were randomly assigned into four groups to take either 1000 mg omega-3 fatty acids containing 180 mg eicosapentaenoic acid (EPA) and 120 mg DHA twice a day + vitamin D placebo (*n* = 30) or 50,000 IU vitamin D every 2 weeks + omega-3 fatty acids placebo (n = 30) or 50,000 IU vitamin D every 2 weeks + 1000 mg omega-3 fatty acids twice a day (*n* = 30) or vitamin D and omega-3 fatty acids placebos (*n* = 30) for 6 weeks. Although, the duration of intervention was 6 weeks, all participants were followed up until the delivery. The placebo capsule contained 500 mg of liquid paraffin. Due to lack of evidence about the appropriate dosage of vitamin D and omega-3 fatty acids for patients with GDM, we used the above-mentioned dose of vitamin D based on a previous study in GDM women [20] and dose of omega-3 fatty acids based on a previous study in patients with end-stage renal disease [21]. The appearance of the placebo capsule was indistinguishable in color, shape, size, and packaging, smell and taste from vitamin D and omega-3 fatty acids capsules. Vitamin D and omega-3 fatty acids capsules were produced by Zahravi Pharmaceutical Company, Tabriz, Iran, that approved by Food and Drug Administration. Compliance with the consumption of supplements and placebos was assessed by examining the tablet containers as well as by the measurement of serum 25-hydroxyvitamin D concentrations with the enzyme-linked immunosorbent assay (ELISA) method. To increase compliance, all participants received short messages on their cell phones every day to remind them about taking the capsules. Randomization assignment was conducted using computer-generated random numbers. Randomization and allocation were concealed from the researchers and participants until the final analyses were completed. Another person, who was not involved in the trial and not aware of random sequences, assigned the subjects to the numbered bottles of capsules. Subjects were requested not to change their routine physical activity or usual dietary intakes throughout the study and not to consume any supplements other than the one provided to them by the investigators as well as not to take any medications that might affect findings during the 6-wk intervention. Dietary macro- and micro-nutrients intakes were determined using the 3-day food records at weeks 0, 3, 5 and 6 of the intervention. Modified Nutritionist-4 software program (First Databank, San Bruno, CA) was used to determine macro- and micro-nutrients intakes. Physical activity was described as metabolic equivalents (METs) in hours per day [22].

### Assessment of anthropometric measurements

Weight and height (Seca, Hamburg, Germany) were determined at baseline and after the treatment in a fasting status without shoes and a minimal clothing state by trained staff. BMI was calculated using the height and weight measurements (weight in kg/ [height in meters] ^2^).

### Outcomes

In the current study, the primary outcomes measurements were inflammatory factors. The secondary outcomes measurements were biomarkers of oxidative stress and pregnancy outcomes.

The measured outcomes of GDM women were biomarkers of inflammation and oxidative, cesarean section, preterm delivery, pre-eclampsia, polyhydramnios and gestational age. The measured outcomes of newborns were macrosomia, newborns’ weight, length and head circumference, apgar score, and newborns’ hyperbilirubinemia, hospitalization and hypoglycemia.

### Assessment of biochemical variables

Five milliliter fasting blood samples were obtained from each patient at the AUMS reference laboratory, Ardabil, Iran, at baseline and the end of the study. Blood samples was collected in 2 separate tubes: 1) one without EDTA to separate the serum, in order to evaluate serum 25-hydroxyvitamin D and high sensitivity C-reactive protein (hs-CRP) concentrations and 2) another one containing EDTA to examine plasma nitric oxide (NO), total antioxidant capacity (TAC), total glutathione (GSH) and malondialdehyde (MDA). Blood samples were immediately centrifuged (Hettich D-78532, Tuttlingen, Germany) at 3500 rpm for 10 min to separate the serum. Serum 25-hydroxyvitamin D concentrations were assessed by an ELISA kit (IDS, Boldon, UK) with inter- and intra-assay coefficient variances (CVs) of 4.4 to 6.6%, respectively. Serum hs-CRP levels were quantified using an ELISA kit (LDN, Nordhorn, Germany) with intra- and inter-assay CVs of 3.4 and 5.1%, respectively. The plasma NO levels by Griess method [23], TAC concentrations using the ferric reducing antioxidant power method developed by Benzie and Strain [24], GSH by the method of Beutler et al. [25] and MDA levels by the thiobarbituric acid reactive substance spectrophotometric test [26] were determined. CVs for plasma NO, TAC, GSH and MDA were lower than 5%.

Newborns’ hyperbilirubinemia was considered when the total serum bilirubin levels were at or above 15 mg/dL (257 mol/L) in infants 25 to 48 h old, 18 mg/dL (308 mol/L) in infants 49 to 72 h old, and 20 mg/dL (342 mol/L) in infants older than 72 h [27].

### Clinical assessment

Polyhydramnios was diagnosed using the sonographic estimation method at post-intervention. On the basis of this measurement, polyhydramnios was defined as an amniotic fluid index (AFI) in excess of 25 cm [16]. Preterm delivery was defined as delivery occurred at <37 weeks of pregnancy and newborn’s macrosomia was defined as birth weight of >4000 g.

### Statistical methods

To calculate the sample size, we used the standard formula suggested for clinical trials by considering type one error (α) of 0.05 and type two error (β) of 0.20 (power = 80%). Based on a previous study [12], we used 1570.5 ng/mL as SD and 1250.0 ng/mL as the difference in mean (d) of hs-CRP concentrations as primary variable. Based on this, we needed 25 patients in each group. Considering a dropouts of 5 patients per group, we calculated to have 30 patients per group.

The Kolmogrov-Smirnov test was used to establish the normal distribution of variables. One-way analysis of variance (ANOVA) was used to detect differences in general characteristics and dietary intakes between the four groups. The changes across three groups were compared using Bonferoni post hoc pair-wise comparisons. The Pearson Chi-square test was used to compare categorical variables. To determine the effects of vitamin D plus omega-3 fatty acids supplementation on biomarkers of inflammation and oxidative stress, we used ANOVA. To assess if the magnitude of the change depended on the baseline values, we adjusted all analyses for the baseline values, age and baseline BMI to avoid the potential bias that might have resulted with ANCOVA test. *P*-values <0.05 were considered statistically significant. All statistical analyses were done using the Statistical Package for Social Science version 17 (SPSS Inc., Chicago, Illinois, USA).

## Results

**Fig. 1:**
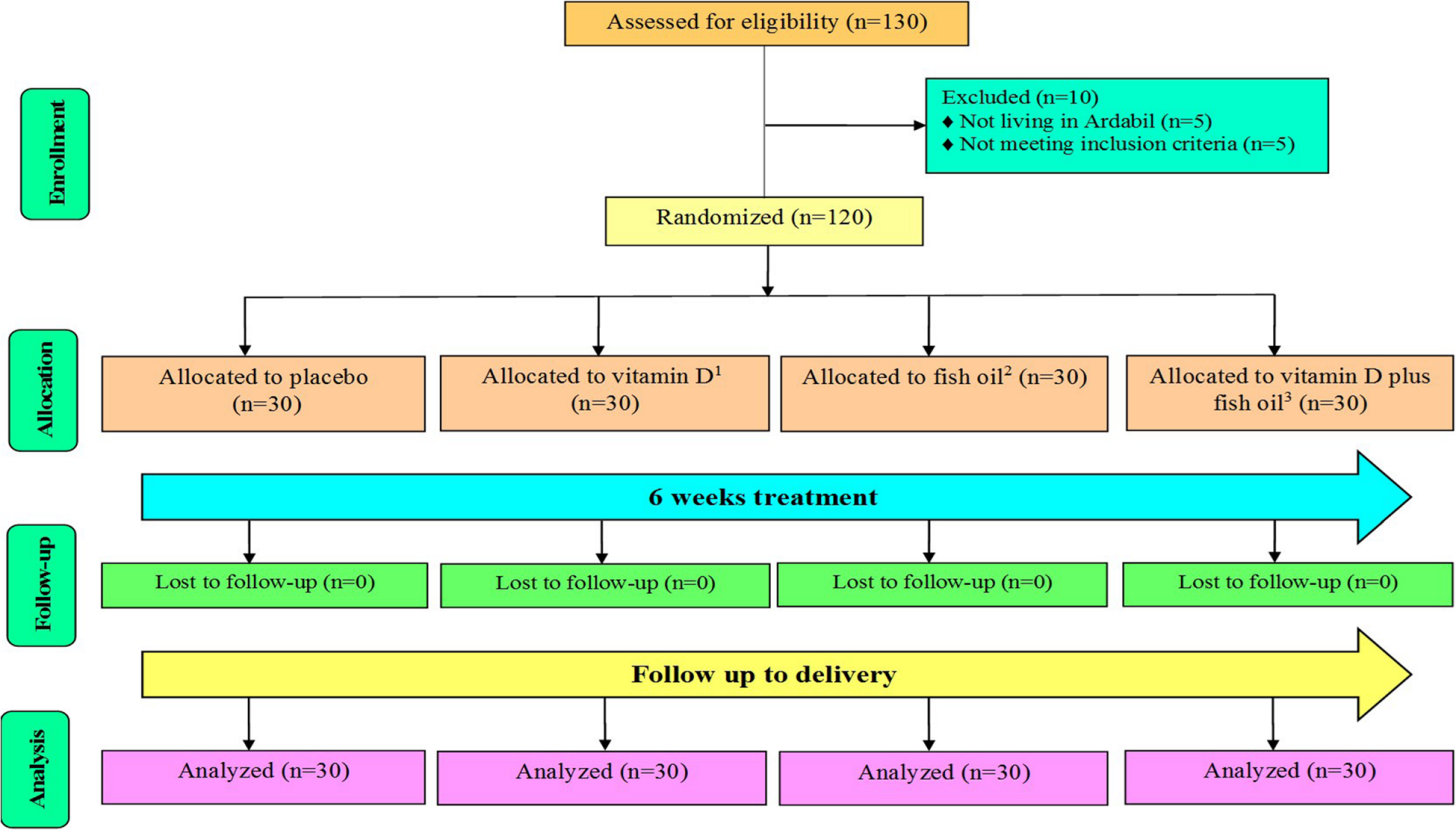
Summary of patient flow diagram, ^1^ Individuals received 50,000 IU vitamin D every 2 weeks plus placebo for omega-3 fatty acids twice a day, ^2^ Individuals received 1000 mg omega-3 fatty acids (180 mg EPA and 120 mg DHA) twice a day plus placebo for vitamin D every 2 weeks, ^3^ Individuals received 50,000 IU vitamin D every 2 weeks plus 1000 mg omega-3 fatty acids twice a day

Initially, 130 subjects were screened; of whom 10 subjects were excluded due to not living in Ardabil (*n* = 5) and not meeting inclusion criteria. In the current study, 120 GDM women [vitamin D (*n* = 30), omega-3 fatty acids (*n* = 30), vitamin D plus omega-3 fatty acids (*n* = 30) and placebo (*n* = 30)] completed the trial (Fig. 1). On average, the rate of compliance in the present study was high, such that higher than 90% of capsules were taken throughout the study in both groups. No side effects were reported following the supplementation of vitamin D and omega-3 fatty acids supplements in women with GDM throughout the study.

Mean age and height of study participants were not statistically different between the four groups (Table 1). Baseline weight and BMI as well as their means after intervention were not significant different comparing the groups.

**Table 1:** General characteristics of study participants^1^

	Placebo (*n* = 30)	Vitamin D ^2^(*n* = 30)	Omega-3^3^ (*n* = 30)	Vitamin D+ omega-3^4^ (*n* = 30)	P^5^
Age (y)	29.2 ± 3.4	29.9 ± 5.0	29.7 ± 3.6	29.9 ± 4.0	0.869
Height (cm)	161.6 ± 4.8	161.5 ± 4.1	161.5 ± 3.4	161.8 ± 3.7	0.985
Weight at study baseline (kg)	75.1 ± 7.7	76.1 ± 12.7	74.3 ± 5.8	77.4 ± 10.2	0.632
Weight at end-of-trial (kg)	77.5 ± 7.7	78.6 ± 12.5	76.7 ± 6.1	80.0 ± 10.0	0.559
Weight change (kg)	2.3 ± 0.7	2.5 ± 1.1	2.4 ± 0.5	2.6 ± 0.6	0.570
BMI at study baseline (kg/m^2^)	28.8 ± 3.4	29.2 ± 5.0	28.5 ± 2.4	29.5 ± 3.8	0.746
BMI at end-of-trial (kg/m^2^)	29.7 ± 3.4	30.1 ± 4.8	29.4 ± 2.5	30.5 ± 3.7	0.683
BMI change (kg/m^2^)	0.9 ± 0.3	1.0 ± 0.4	0.9 ± 0.2	1.0 ± 0.2	0.592

^1^Data are means ± SDs

^2^Receiving 50,000 IU vitamin D every 2 weeks plus placebo for omega-3 fatty acids twice a day

^3^Receiving 1000 mg omega-3 fatty acids (180 mg EPA and 120 mg DHA) twice a day plus placebo for vitamin D every 2 weeks

^4^Receiving 50,000 IU vitamin D every 2 weeks plus 1000 mg omega-3 fatty acids twice a day

^5^Obtained from ANOVA test

Based on the 3-day dietary records obtained throughout the intervention, no statistically significant change was seen between the four groups in terms of dietary intakes of macro- and micronutrients (Data not shown).

After 6 weeks of intervention, co-supplementation with vitamin D and omega-3 fatty acids led to a significant increase in serum 25-hydroxyvitamin D values (+19.5 ± 3.2 vs. +18.1 ± 4.4, +0.7 ± 2.8 and −0.2 ± 1.7 ng/mL, respectively, *P* < 0.001) compared with vitamin D, omega-3 fatty acids and placebo **(**Table 2 **).** In addition, subjects who received vitamin D plus omega-3 fatty acids supplements compared with vitamin D, omega-3 fatty acids and placebo had significantly decreased serum hs-CRP (−2.0 ± 3.3 vs. −0.8 ± 4.4, −1.3 ± 2.4 and +0.9 ± 2.7 mg/L, respectively, *P* = 0.008), plasma MDA (−0.5 ± 0.5 vs. -0.2 ± 0.5, −0.3 ± 0.9 and +0.5 ± 1.4 μmol/L, respectively, *P* < 0.001), and increased plasma TAC (+92.1 ± 70.1 vs. +55.1 ± 123.6, +88.4 ± 95.2 and +1.0 ± 90.8 mmol/L, respectively, *P* = 0.001) and GSH levels (+95.7 ± 86.7 vs. +23.0 ± 62.3, +30.0 ± 66.5 and −7.8 ± 126.5 μmol/L, respectively, P = 0.001). Co-supplementation with vitamin D and omega-3 fatty acids had no significant effect on plasma NO levels.

**Table 2:** The effect of vitamin D plus omega-3 fatty acids supplementations on biomarkers of inflammation and oxidative in gestational diabetes patients^1^

	Placebo (*n* = 30)	Vitamin D^2^ (*n* = 30)	Omega-3^3^ (*n* = 30)	Vitamin D+ omega-3^4^(*n* = 30)	P^5^
Serum 25-OH-vitamin D (ng/mL)					
Baseline	14.9 ± 3.2	13.6 ± 3.7	15.6 ± 4.0	14.2 ± 2.9	0.156
End	14.7 ± 3.2	31.7 ± 6.8	16.3 ± 4.6	33.8 ± 4.6	<0.001
Change	-0.2 ± 1.7	18.1 ± 4.4^b^	0.7 ± 2.8	19.5 ± 3.2^b^	<0.001
hs-CRP (mg/L)					
Baseline	7.2 ± 3.9	7.2 ± 6.4	8.0 ± 6.2	8.3 ± 4.6	0.815
End	8.1 ± 4.0	6.4 ± 3.9	6.7 ± 5.9	6.3 ± 4.7	0.407
Change	0.9 ± 2.7	-0.8 ± 4.4	−1.3 ± 2.4	−2.0 ± 3.3^a^	0.008
NO (μmol/L)					
Baseline	51.1 ± 13.0	54.1 ± 4.9	46.9 ± 4.9	46.1 ± 5.2	<0.001
End	52.5 ± 15.4	60.0 ± 7.9	51.5 ± 7.3	48.4 ± 5.1	<0.001
Change	1.4 ± 11.4	5.9 ± 9.0	4.6 ± 9.2	2.3 ± 7.5	0.230
TAC (mmol/L)					
Baseline	748.1 ± 109.7	807.2 ± 96.6	811.0 ± 54.3	906.4 ± 73.9	<0.001
End	749.1 ± 91.2	862.3 ± 91.9	899.4 ± 112.8	998.5 ± 82.1	<0.001
Change	1.0 ± 90.8	55.1 ± 123.6	88.4 ± 95.2^a^	92.1 ± 70.1^a^	0.001
GSH (μmol/L)					
Baseline	491.8 ± 173.5	456.6 ± 67.8	532.5 ± 49.6	518.3 ± 64.2	0.020
End	484.0 ± 161.2	479.6 ± 59.7	562.5 ± 57.8	594.2 ± 60.9	<0.001
Change	−7.8 ± 126.5	23.0 ± 62.3	30.0 ± 66.5	75.9 ± 86.7^a^	0.005
MDA (μmol/L)					
Baseline	3.2 ± 1.0	2.7 ± 0.4	3.5 ± 0.8	3.0 ± 0.5	<0.001
End	3.7 ± 1.6	2.5 ± 0.3	3.2 ± 0.4	2.5 ± 0.3	<0.001
Change	0.5 ± 1.4	−0.2 ± 0.5^a^	−0.3 ± 0.9^a^	−0.5 ± 0.5^a^	<0.001

^1^Data are means ± SDs

^2^Receiving 50,000 IU vitamin D every 2 weeks plus placebo for omega-3 fatty acids twice a day

^3^Receiving 1000 mg omega-3 fatty acids (180 mg EPA and 120 mg DHA) twice a day plus placebo for vitamin D every 2 weeks

^4^Receiving 50,000 IU vitamin D every 2 weeks plus 1000 mg omega-3 fatty acids twice a day

^5^Obtained from ANOVA test

GSH, glutathione; hs-CRP, high-sensitivity C-reactive protein; MDA, malondialdehyde; NO, nitric oxide; TAC, total antioxidant capacity

^a^Significant difference with the placebo group

^b^Significant difference with the placebo and omega-3 fatty acids groups

There was a significant difference in baseline levels of plasma NO (*P* < 0.001), TAC (*P* < 0.001), GSH (*P* = 0.02) and MDA (*P* < 0.001) between the four groups. When we adjusted the analysis for baseline values of biochemical parameters, age and baseline BMI, findings did not change except plasma NO levels (0.020) **(**Table 3 **).**

**Table 3:** Adjusted changes in metabolic variables in gestational diabetes patients that received either vitamin D plus omega-3 fatty acids, omega-3 fatty acids and vitamin D supplements or placebo^1^

	Placebo (*n* = 30)	Vitamin D ^2^ (*n* = 30)	Omega-3^3^ (*n* = 30)	Vitamin D+ omega-3^4^ (*n* = 30)	P^5^
Serum 25-OH-vitamin D (ng/mL)	−0.2 ± 0.6	18.2 ± 0.6	0.7 ± 0.6	19.5 ± 0.6	<0.001
hs-CRP (mg/L)	0.8 ± 0.5	−1.0 ± 0.5	−1.2 ± 0.5	−1.9 ± 0.5	0.004
NO (μmol/L)	1.9 ± 1.6	7.9 ± 1.6	3.3 ± 1.6	1.0 ± 1.6	0.020
TAC (mmol/L)	−35.5 ± 17.1	49.1 ± 15.9	85.5 ± 15.8	137.6 ± 17.8	<0.001
GSH (μmol/L)	−12.4 ± 14.2	5.6 ± 14.5	45.2 ± 14.4	83.8 ± 14.3	<0.001
MDA (μmol/L)	0.5 ± 0.1	−0.4 ± 0.2	−0.1 ± 0.2	−0.5 ± 0.1	<0.001

^1^All values are means ± SEs. Values are adjusted for baseline values of parameters of biochemical, age and baseline BMI

^2^Receiving 50,000 IU vitamin D every 2 weeks plus placebo for omega-3 fatty acids twice a day

^3^Receiving 1000 mg omega-3 fatty acids (180 mg EPA and 120 mg DHA) twice a day plus placebo for vitamin D every 2 weeks

^4^Receiving 50,000 IU vitamin D every 2 weeks plus 1000 mg omega-3 fatty acids twice a day

^5^Obtained from ANCOVA test

GSH, glutathione; hs-CRP, high-sensitivity C-reactive protein; MDA, malondialdehyde; NO, nitric oxide; TAC, total antioxidant capacity

Vitamin D and omega-3 fatty acids co-supplementation, compared with vitamin D, omega-3 fatty acids and placebo, resulted in lower incidences of newborns’ hyperbilirubinemiain (10.0% vs. 20.0%, 33.3% and 40.0%, respectively, *P* = 0.037) and newborns’ hospitalization (10.0% vs. 20.0%, 33.3% and 40.0%, respectively, *P* = 0.037), but did not affect other pregnancy outcomes **(**Table 4 **).**

**Table 4:** The association of vitamin D plus omega-3 fatty acids supplementation with pregnancy outcomes

	Placebo group (*n* = 30)	Vitamin D ^2^ (*n* = 30)	Omega-3^3^ (*n* = 30)	Vitamin D+ omega-3^4^ (*n* = 30)	P^5^
Cesarean section (%)	11 (36.7)	9 (30.0)	9 (30.0)	8 (26.7)	0.863^†^
Preterm delivery (%)	1 (3.3)	0 (0.0)	0 (0.0)	0 (0.0)	0.388^†^
Pre-eclampsia (%)	3 (10.0)	3 (10.0)	2 (6.7)	3 (10.0)	0.960^†^
Polyhydramnios (%)	2 (6.6)	2 (6.6)	2 (6.6)	2 (6.6)	>0.999^†^
Macrosomia > 4000 g (%)	5 (16.7)	2 (6.7)	3 (10.0)	2 (6.7)	0.525^†^
Gestational age (weeks)	39.1 ± 1.1	39.2 ± 1.0	39.0 ± 1.4	39.3 ± 0.9	0.658
Newborns’ weight (g)	3323.0 ± 407.1	3308.3 ± 604.0	3475.0 ± 395.2	3311.7 ± 502.2	0.480
Newborns’ length (cm)	50.4 ± 1.1	50.2 ± 3.0	51.3 ± 2.1	49.9 ± 2.7	0.103
Newborns’ head circumference (cm)	35.7 ± 1.3	35.9 ± 2.9	35.5 ± 1.4	35.3 ± 2.7	0.744
1- min Apgar score	8.8 ± 0.4	8.7 ± 0.4	8.7 ± 0.4	8.8 ± 0.4	0.779
5- min Apgar score	9.8 ± 0.4	9.7 ± 0.4	9.7 ± 0.4	9.8 ± 0.4	0.779
Newborns’ hyperbilirubinemia (%)	12 (40.0)	6 (20.0)	10 (33.3)	3 (10.0)	0.037^†^
Newborns’ hospitalization (%)	12 (40.0)	6 (20.0)	10 (33.3)	3 (10.0)	0.037^†^
Newborns’ hypoglycemia (%)	6 (20.0)	4 (13.3)	5 (16.7)	4 (13.3)	0.876^†^

Values are means ± SDs for continuous measures and are number (%) for dichotomous variables

^†^Obtained from Pearson Chi-square test

^2^Receiving 50,000 IU vitamin D every 2 weeks plus placebo for omega-3 fatty acids twice a day

^3^Receiving 1000 mg omega-3 fatty acids (180 mg EPA and 120 mg DHA) twice a day plus placebo for vitamin D every 2 weeks

^4^Receiving 50,000 IU vitamin D every 2 weeks plus 1000 mg omega-3 fatty acids twice a day

^5^Obtained from ANOVA test

## Discussion

To our knowledge, this study is the first report of the effects of vitamin D and omega-3 fatty acids co-supplementation on biomarkers of inflammation, oxidative stress and pregnancy outcomes among women with GDM. We found that vitamin D and omega-3 fatty acids co-supplementation for 6 weeks to GDM women had beneficial effects on maternal serum hs-CRP, plasma TAC, GSH, MDA levels, and newborns’ hyperbilirubinemiain and hospitalization, but did not influence plasma NO levels and other pregnancy outcomes. In the current study, we hypothesized that combined therapy with vitamin D and omega-3 fatty acids in GDM women may work better than a single supplementation alone. In addition, vitamin D and omega-3 fatty acids co-supplementation might have a strong synergistic effect on biomarkers of inflammation, oxidative stress and pregnancy outcomes. In a study by Baidal et al. [28], it was observed that combined omega-3 fatty acids and vitamin D3 therapy was well tolerated and had beneficial effects on beta-cell function in patients with onset type 1 diabetes. Gurol et al. [29] also demonstrated that omega-3 fatty acids and vitamin D had a synergistic effect on glycemia in islet transplantation. Furthermore, omega-3 fatty acids supplementation may result in increased circulating levels of vitamin D. In a study by An et al. [30], it was seen that 1,25(OH)_2_D levels significantly increased in dialysis patients compared to baseline after 3 months of omega-3 fatty acids supplementation without vitamin D. Omega-3 fatty acids intake may also overcome the inverse association of vitamin D deficiency with inflammation [31]. As most patients of our study had vitamin D deficiency, decreased inflammatory markers may improve pregnancy outcomes.

The current study showed that vitamin D and omega-3 fatty acids co-supplementation to GDM women for 6 weeks resulted in a significant reduction in serum hs-CRP, but did not affect plasma NO levels compared with other groups. Although few studies have assessed beneficial effects of vitamin D or omega-3 fatty acids supplementation on inflammatory cytokines, to the best of our knowledge, data on the effects of vitamin D and omega-3 fatty acids co-supplementation on inflammatory cytokines are scarce. We have previously demonstrated that supplementation with 50,000 IU of vitamin D every 3 weeks [11] or 1000 mg/day of omega-3 fatty acids [12] for 6 weeks to GDM women had beneficial effects on few pregnancy outcomes. Likewise, in some studies, vitamin D supplementation in patients with a history of myocardial infarction and elderly women with vitamin D deficiency was associated with a significant reduction in circulating levels of CRP [32, 33]. The administration of fish oil at a dosage of 3 g/day to men with coronary artery disease for 8 weeks decreased hs-CRP levels [34]. However, in GDM women, vitamin D supplementation at a dosage of 50,000 IU every 2 weeks for 2 months had no significant effect on hs-CRP levels [20]. In addition, short-term omega-3 fatty acids supplementation (3 g/day for 8 weeks) in patients with T2DM could not affect CRP levels [35]. Increasing production of inflammatory cytokines in GDM would result in increased adiposity at birth [36] as well as predisposes the newborn to become overweight and develop metabolic diseases including glucose intolerance, metabolic syndrome, and cardiovascular disease [37, 38]. Less production of parathyroid hormone after vitamin D intake [39] might result in the reducing production of inflammatory factors including CRP. Furthermore, increased gene expression of peroxisome proliferator-activated receptors by omega-3 inhibits the activation of NF-кB [18], which in turn can decrease the production of inflammatory cytokines.

We demonstrated that vitamin D plus omega-3 fatty acids supplementation in GDM women for 6 weeks led to significant increases in plasma TAC and GSH concentrations, and a significant decrease in plasma MDA levels compared with other groups. Similarly our findings were observed following high-dose vitamin D supplementation (50,000 IU every 2 weeks) increased TAC and GSH concentrations in patients with GDM [40]. In addition, supplementation with vitamin D at a dosage of 200,000 IU significantly increased TAC levels in elderly subjects with vitamin D insufficiency [33]. Vitamin D supplementation at a dosage of 100,000 IU monthly for 3 months to overweight and obese subjects also decreased oxidative stress mediators of arterial stiffness [41]. On the other hand, a significant beneficial effect was seen following supplementation with 400 mg/day of DHA for 2 weeks on platelet function and oxidative stress in patients with T2DM [42]. Furthermore, fish oil supplementation significantly decreased biomarkers of oxidative stress after a single bout of eccentric exercise in healthy man [43]. However, supplementation of fish oil to patients with Alzheimer’s disease for 6 months did not affect biomarkers of oxidative stress [44]. Oxidative stress imbalance in GDM is important in the pathophysiological processes involved in vascular diseases, such as diabetes, hypertension and cardiovascular diseases [45]. Vitamin D intake can improve oxidative stress through its antioxidant properties [46], and decreasing production of reactive oxygen species and pro-inflammatory cytokines [47]. In addition, observed beneficial effects on biomarkers of oxidative stress by omega-3 fatty acids may be mediated by their anti-inflammatory properties [48].

It must be kept in mind that in the current study, vitamin D and omga-3 fatty acids co-supplementation may have an indirect role in newborns’ hyperbilirubinemia and hospitalization due to their effects on improved biomarkers of inflammation and oxidative stress. In a study by Kalra et al. [49], it observed that supplementation with one dose of 1500 μg vitamin D3 in the second trimester or two doses of 3000 μg vitamin D3 each in the second and third trimesters resulted in improved liver enzyme levels in cord blood of the infants. In addition, fish oil supplementation (0.2 g/kg body weight daily) for 5 days in postoperative cancer subjects was associated with significant reductions of bilirubin and liver enzymes levels [50]. However, supplementation with 25 mg/day of ergocalciferol to pregnant women did not affect mean birth weight [51, 52]. Moreover, supplementation with 600 mg/day of DHA in the last half of gestation resulted in overall greater gestation duration and infant size [53]. Active form of vitamin D induces vitamin D receptors, which in turn act as a receptor for secondary bile acids, such as lithocholic acid and 3-ketocholanic acid, and leads to their catabolism via induction of cytochrome 3A enzymes [17, 54].

Few limitations must be considered in the interpretation of our findings. The main limitation of our study is the lack of measurements of circulating levels of EPA, DHA and other omega-3 fatty acids at baseline and after the 6-week intervention due to budget limitations. In addition, further studies are needed to assess the gene expression related to inflammation and oxidative stress to explore the plausible mechanism and confirm our findings.

## Conclusions

Overall, vitamin D and omega-3 fatty acids co-supplementation for 6 weeks among GDM women had beneficial effects on some biomarkers of inflammation, oxidative stress and pregnancy outcomes.

## Abbreviations

AFI: Amniotic fluid index
BMI: Body mass index
CAD: Coronary artery disease
DHA: Docosahexaenoic acid
ELISA: Enzyme-linked immunosorbent assay
EPA: Eicosapentaenoic acid
GDM: Gestational diabetes mellitus
GSH: Glutathione
hs-CRP: High-sensitivity C-reactive protein
MDA: Malondialdehyde
METs: Metabolic equivalents
NF-кB: Nuclear factor-κB
NO: Nitric oxide
T2DM: Type 2 diabetes mellitus
TAC: Total Antioxidant Capacity
